# Traditional Chinese medicine for management of recurrent and refractory Crohn disease: A case report

**DOI:** 10.1097/MD.0000000000015148

**Published:** 2019-04-12

**Authors:** Hezheng Lai, Kang Wang, Qing Dong, Xiaoshu Zhu, Xiaoke Li, Shuo Qi

**Affiliations:** a Department of Thyroid; b Department of Hematology and Oncology; c Department of Gastroenterology, Dongzhimen Hospital; d Department of Tuina and Physiotherapy, Dongfang Hospital, Affiliated to Beijing University of Chinese Medicine, Beijing, China; e School of Science and Health, Western Sydney University, Campbelltown, New South Wales; f The Chinese Medicine Center, Collaboration Between Beijing University of Chinese Medicine and Western Sydney University; g NICM Health Research Institute, Western Sydney University, Sydney, New South Wales, Australia.

**Keywords:** acupuncture, Crohn disease, traditional Chinese medicine

## Abstract

**Introduction:**

Crohn disease (CD) is a chronic relapsing systemic inflammatory disease afflicting the gastrointestinal system with a high morbidity. There has been increasing clinical interest in traditional Chinese medicine (TCM) treatment for CD. This report aims to present patient outcome of therapeutic management using TCM in combination with pharmacologic therapy.

**Patient concerns:**

A 53-year-old woman with a more than 23-year history of chronic indigestion, reflux, abdominal pain, and excessive diarrhea, and a more than 21-year history of recurrent refractory CD. The condition had been managed for 21 years with ongoing pharmacologic therapy, and surgical intervention; however, with poor therapeutic effect.

**Diagnosis:**

In this study, the diagnosis of CD was based on integrating patient symptoms and radiologic findings and biopsy results. The patient had no differential diagnosis.

**Interventions:**

The patient received acupuncture treatment at an approximate frequency of once per week for a total of 21 sessions until November 5, 2018. The patient also received Chinese herbal medicine (CHM) on an as-needed basis to manage her symptoms.

**Outcomes:**

Patient reported symptoms of chronic indigestion, reflux, abdominal pain, and excessive diarrhea were substantially improved by combined TCM and pharmacologic therapy intervention, while the dosage of her medication was reduced.

**Conclusion:**

Following acupuncture treatment, improvements of subjective symptoms: chronic indigestion, reflux, abdominal pain, and excessive diarrhea, were observed. CHM provided immediate relief of indigestion, reflux, and abdominal pain. TCM may be a potential therapeutic strategy to manage clinical symptoms of CD, if this is proven useful in future RCT studies.

## 1 Introduction

Crohn disease (CD) is a chronic relapsing systemic inflammatory disease afflicting the gastrointestinal system with a high morbidity.^[1,2]^ It is a type of inflammatory bowel disease (IBD) that may involve any portion of the gastrointestinal tract and numerous extraintestinal sites.^[3,4]^ Presenting symptoms are often variable and frequently include abdominal pain, fever, diarrhea with passage of blood or mucous, or both, nausea, vomiting, and weight loss.^[2,5]^ CD may cause complications including large and small bowel obstructions, fistulas and intra-abdominal abscesses, and perianal disease.^[3,6]^ Quality of life is often impacted.^[7]^

Current therapeutic goals for CD are to induce and maintain a steroid-free disease remission, decrease the risk of complications and surgery, prevent disease progression, and improve overall quality of life.^[5,8,9]^ Disease management is usually with pharmacologic therapy. The choice of medication depends on disease severity and response to previous therapies. The most commonly used drugs in CD are corticosteroids, immunosuppressants (thiopurines [azathioprine and mercaptopurine] and methotrexate), biologicals (anti-TNF (tumor necrosis factor) [infliximab, adalimumab, and certolizumab pegol], and anti-adhesion molecules (vedolizumab).^[9]^ While anti-TNF therapy appears to be the most effective therapy, used alone or in combination with an immunomodulator to induce and maintain remission in moderate to severe CD,^[5]^ it was shown to double the risk of opportunistic infections^[10]^ and might increase the risk of melanoma skin cancer.^[11]^

Furthermore, a large challenge associated with CD is that approximately after 20 years of disease activity 80% of patients will require a surgery^[5]^ and approximately 30% of patients require major abdominal surgery within 5 years of diagnosis.^[12]^ As surgery is not curative for CD, patients often require multiple surgeries over their lifetime once stricturing and/or fistulizing complications occur.^[13]^ The clinical recurrence rate may be 30% within 2 years of surgery.^[3]^ Many patients still require ongoing lifelong pharmacologic therapy even after surgery for disease recurrence and suffer poor quality of life due to continuing symptom burden.^[5]^

Acupuncture is a primary therapy used in traditional Chinese medicine (TCM) and has been widely used for gastrointestinal diseases.^[14]^ Findings from randomized controlled trials suggest that acupuncture combined with moxibustion may help to improve CD symptoms and patient quality of life.^[15,16]^ However, to date, no study has reported the improvements in effectiveness with acupuncture as the primary therapy. The current case provides clinicians with further clinical evidence of the use of acupuncture as a therapeutic strategy in the management of this recurrent and refractory disease.

## 2 Case report

A 53-year-old woman with a more than 23-year history of chronic indigestion, reflux, abdominal pain and excessive diarrhea, and a more than 21-year history of CD presented to the clinic on December 11, 2017. The patient had no specific medical conditions within her family history. She had no prior history of alcohol consumption and she was a nonsmoker.

The patient first experienced symptoms of persistent diarrhea and abdominal pain in 1994. In 1997, she underwent comprehensive testing including stool cultures, gastroscopy, colonoscopy, and small bowel biopsy and numerous blood tests, which ultimately confirmed very active small bowel CD and a small patch of colitis at her terminal ilium, palpable hemorrhoids, lactase deficiency, and shallow duodenal ulcers. Thorough treatment of the duodenal ulcers and a lactose-free diet made no difference to her complaints. Stools were greater than 10 per day without medication and often 1 or 2 at night.

CD was managed with pharmacologic therapy mesalazine (500 mg Bid Po), prednisone (75 mg Qd Po to induce remission and 5 mg Qd Po as ongoing maintenance dosage), and azathioprine (50 mg Bid Po). Although medication helped the patient to return to work and resume her daily life, the condition was not well controlled. She continued to suffer from blockages and symptoms of pain and vomiting, for which she required frequent hospitalization and in 2005 she underwent a bowel resection. Pharmacologic therapy was continued after surgery and helped to maintain symptom remission; however, the patient continued to experience blockages, accompanied symptoms of pain and vomiting, which occurred on a monthly frequency. As a result, in 2013, the patient received a second bowel resection and repair of strictures.

Following surgery in 2013, pharmacologic therapy was continued to manage the patient's symptoms mesalazine (500 mg Bid Po), prednisone (increased to 100 mg Qd Po to induce remission and 5 mg Qd Po as ongoing maintenance dosage), and azathioprine (increased to 50 mg Tid Po). Due to long-term side effects of the drug, prednisone was stopped in May 2017.

The patient presented with chronic indigestion, reflux, abdominal pain characterized by sensation of heat, and excessive diarrhea which caused her discomfort and decreased the quality of her daily life. The symptoms were chronic and were also aggravated by stress. She experienced abdominal pain and diarrhea 5 to 6 times a day. She continued to be managed using pharmacologic therapy azathioprine (50 mg Tid Po) and mesalazine (500 mg Tid Po). At the start of treatment, in addition to her daily regular medication, she was also taking Mylanta (Infirst Healthcare Inc., Westport, CT) (magnesium hydroxide 800 mg, aluminum hydroxide-dried 800 mg), and Gastro-Stop (Aspen Pharmacare Australia Pty Ltd, Victoria, Australia) (loperamide hydrochloride 4 mg Bid Po). The therapeutic effect was nevertheless poor; reflux and indigestion were unable to be relieved, and only minimal reduction was seen in diarrhea. Medication was unable to make apparent improvement to her symptom spectrum or improve quality of life.

The patient received acupuncture treatment on December 11, 2017. She was treated with acupuncture at an approximate frequency of once per week for a total of 21 sessions until November 5, 2018. The acupuncture points selected in this case are generally used to treat digestive and gastrointestinal diseases. The patient was in a supine position during acupuncture treatment. After the skin at the site of needle insertion was sterilized, disposable sterilized acupuncture needles were inserted at Ququan (LV 8), Quchi (LI 11), Zhongwan (CV12), Qihai (CV6), Zusanli (ST36), and Sanyinjiao (SP6). All points were needled bilaterally except for CV12, CV6, and LV8 which was needled on the left side. Deqi (the patient's feeling of heaviness and dull aching sensation due to the needles) was obtained at all points. Needles were retained for another 20 minutes without further stimulation. On February 5, 2018, the patient was commenced on granule-form Chinese herbal medicine (CHM) formula Tong Xie Yao Fang for 2 weeks.

During the intervention, the patient did not receive any further treatment from any other clinics or hospitals. In addition to receiving acupuncture treatment she continued taking her regular medications azathioprine (50 mg Tid Po) and mesalazine (500 mg Tid Po), Mylanta (magnesium hydroxide 800 mg, aluminum hydroxide-dried 800 mg), and Gastro-Stop (loperamide hydrochloride 4 mg Bid Po).

The therapeutic effect of acupuncture treatment was positive. Over the duration of the treatment period, the patient experienced marked improvement in her symptoms. By March 2018, the patient's symptoms were well managed; flare-ups of diarrhea, indigestion, reflux, and abdominal pain, occurred sporadically, and only due to diet-related factors. From March 2018, patient reported reduction of Gastro-Stop from 2 tablets per day to 1, reduction of abdominal pain and diarrhea from an average of 5 to 6 times a day to an average of 3 times a day. She reported that her symptoms were no longer aggravated during times of stress. She continued to receive acupuncture on a monthly basis from August 13, 2018 until November 5, 2018. At the end of the treatment period she reported 100% resolution in all of her symptoms: chronic indigestion, reflux, abdominal pain, and diarrhea.

Furthermore, the therapeutic effect of the CHM formula Tong Xie Yao Fang was also positive. The patient reported provided immediate relief of indigestion, reflux, and abdominal pain after taking CHM. She continued to take the formula only on an as-needed basis, whenever she experienced flare-ups of indigestion, reflux, and abdominal pain caused by diet-related factors.

## 3 Discussion and conclusions

Clinical diagnosis of CD is established through integrating patient symptoms and radiologic and endoscopic findings.^[2,9,14]^ Pathology can help to confirm diagnosis. Common pathological findings include epithelioid granuloma, transmural lymphoid aggregates, pyloric gland metaplasia, chronic focal, patchy, discontinuous, and transmural inflammatory infiltrate, and goblet cell preservation.^[9]^ Typical endoscopic findings include segmental inflammation, apthoid, and longitudinal and serpiginous ulcerations.^[9]^ Typical laboratory findings include thrombocytosis, increased acute phase proteins (particularly C-reactive protein), and anemia.^[9]^

After diagnosis is established, the pharmacologic treatment is determined by the phenotype, disease activity, comorbidities, and other individual characteristics of the drug and patient. Disease activity, severity, extent and behavior, and presence of complications such as strictures or fistulas are assessed using cross-sectional imaging such as computed tomography-enterography or magnetic resonance-enterography.^[2,9]^ Patients are classified by phenotype according to the Montreal classification: inflammatory, stricturing, and fistulizing.^[2,9]^ Inflammatory CD is characterized by inflammation of the gastrointestinal tract with no evidence of stricturing or fistulizing disease.^[5]^ Stricturing disease is characterized by fibrosis and luminal narrowing caused by inflammation of the gastrointestinal tract.^[5]^ Only surgical intervention is able to reverse fibrostenotic changes once they occur. Fistulizing CD is characterized by development of a sinus or fistulous tract caused by ongoing transmural inflammation.^[5]^ Disease severity is determined by the combination of the effect of disease in the individual patient, the cumulative complications and surgical resections, the disability produced by disease, the inflammatory burden of disease, and the disease course.^[9]^

Currently, no available treatment corrects the underlying genetic basis of this chronic illness, so patients with CD require ongoing follow-up due to risk of flare-ups and long complications.^[2]^ Long-term treatment is recommended for CD with the goal to induce and maintain a steroid-free clinical and endoscopic remission (mucosal healing), to stop the naturally progressive destructive course of disease that results in intestinal failure, related complications and surgery, and to improve the patient's overall quality of life.^[2]^ Cross-sectional imaging is used during ongoing follow-up to assess disease activity, complications, and response to therapies. Colonoscopy is used to assess mucosal healing, to monitor disease activity through colorectal neoplasia surveillance, and to manage complications, such as strictures.^[9]^

The most common treatment strategy involves the use of a fast-acting, short-term use agent (i.e., systemic corticosteroids or anti-TNF) to induce rapid symptom relief and to control disease progression and achieve long-term maintenance by combining with thiopurines.^[2]^ Current conventional pharmacologic agents cause side effects; the clinical priority is to achieve a balance between efficacy with side effects and long-term complications of the disease.

Systemic corticosteroids are used to induce remission of moderate to severe CD; however, review studies determine they are not an effective maintenance agent^[5,17,18]^ as they increase the risk of serious infection and mortality in patients with moderate to severe CD.^[5]^ Currently, anti-TNF therapy is regarded the most effective therapy for moderate to severe CD when used alone or in combination with an immunomodulator to induce and maintain remission; however, it carries risk of malignancies and severe infections due to their effects on the immune system.^[4]^ Immunosuppressants thiopurines (azathioprine and mercaptopurine) have been found by a Cochrane review of the literature to be no better than placebo for induction of remission or clinical improvement in ongoing active CD.^[19,20]^ While mesalamine has been widely used in the past, it is currently recommended against use by the American College of Gastroenterology and by European treatment guidelines,^[21]^ and studies have found it ineffective to induce or maintain disease remission.^[22]^ Ustekinumab, an interleukin (IL)-12/IL-23 inhibitor, has been recently approved for use and has been shown to be as effective as anti-TNF therapy at inducing and maintaining remission in moderate to severe CD.^[23]^ However, an Randomized Controlled Trial (RCT) study found no improvement in mucosal healing in comparison with placebo, while data on the quality of life are not yet available.^[24]^ Long-term clinical application of these current primary pharmacologic treatments is limited by poor efficacy, association with significant adverse effects, and likelihood of recurrence after dose reduction or discontinuation.^[16]^

Due to the chronic recurrent and refractory nature of CD and the negative side effects of many of the conventional therapies, many patients turn to complementary and alternative medicine (CAM) to manage their symptoms. TCM therapy acupuncture is a CAM that has been shown to be effective for treating various pain and gastrointestinal disorders.^[25]^ There has been increasing clinical interest in acupuncture treatment for IBD, and animal studies have shown that acupuncture therapy combined with moxibustion can effectively control bowel inflammation by providing multitargeted regulation of the body's physiological balance.^[26]^ While RCT studies^[15,27]^ show evidence for acupuncture and moxibustion in improving disease activity scores in IBD (in active CD and ulcerative colitis, respectively), they did not demonstrate improvement through measures for quality of life or symptom scores. RCT study found that acupuncture offers an additional therapeutic benefit in patients with mild to moderately active CD, on top of a placebo effect.^[15]^ A systematic review and meta-analysis of 43 RCTs^[28]^ concluded that acupuncture and moxibustion therapy demonstrate better efficacy than oral sulfasalazine in treating IBD. Acupuncture in combination with moxibustion may be beneficial for the treatment of IBD in particular CD; however, its effects have not been formally studied as a standalone therapy in conjunction with pharmacologic intervention.

The positive outcome in the treatment of recurrent and refractory CD in this case using acupuncture in conjunction with standard care pharmacologic drugs is unique evidence introduced to the medical research for the first time. This case demonstrates that acupuncture was successful in treating the patient's refractory symptoms of CD (chronic indigestion, reflux, abdominal pain, and excessive diarrhea), suffered with ongoing recurrence over 23 years and unsuccessfully managed using standard care. Acupuncture not only achieved total symptom resolution, but also achieved dosage reduction of her standard care pharmacologic drugs (name drugs) while achieving good clinical outcome and improved quality of life in particular diseases such as CD,^[15]^ ulcerative colitis,^[27]^ irritable bowel syndrome,^[29]^ and functional dyspepsia.^[30]^ According to TCM theory, spleen deficiency and excess dampness is the common pattern of CD, often accompanied with symptoms of kidney weakness and liver stagnation.^[16]^ In this case, we used acupuncture points Ququan (LV 8), Quchi (LI 11), Zhongwan (CV12), Qihai (CV6), Zusanli (ST36), and Sanyinjiao (SP6) and CHM herbs for this pattern to manage the symptoms.

CD is frequently encountered clinically. Pharmacologic treatment is the primary treatment, while surgery is necessary to resolve complications. However, some cases of CD are not well managed using pharmacologic treatment and surgery. Through this case, TCM was used in combination with pharmacologic treatment and achieved positive outcomes. TCM is a potential therapeutic strategy to manage clinical symptoms of CD.

## Acknowledgments

Authors thank everyone that contributed to this study.

## Author contributions

**Supervision:** Xiaoshu Zhu.

**Writing – original draft:** Hezheng Lai.

**Writing – review & editing:** Kang Wang, Qing Dong, Xiaoke Li, Shuo Qi.

Shuo Qi orcid: 0000-0001-7559-4606.
